# Cost-effectiveness of a national population-based screening program for type 2 diabetes: the Brazil experience

**DOI:** 10.1186/s13098-015-0090-8

**Published:** 2015-10-31

**Authors:** Cristiana M. Toscano, Xiaohui Zhuo, Kumiko Imai, Bruce B. Duncan, Carísi A. Polanczyk, Ping Zhang, Michael Engelgau, Maria Inês Schmidt, For the CNDDM Working Group

**Affiliations:** Federal University of Rio Grande do Sul, Porto Alegre, RS Brazil; Centers for Disease Control and Prevention, Atlanta, GA USA; Federal University of Goiás (UFG), Rua T-62, 595, Apto. 201, Goiânia, GO 74223-180 Brazil; National Institutes of Health, Bethesda, Maryland USA; UNICEF, New York City, New York USA

## Abstract

**Background:**

The cost-effectiveness of screening for type 2 diabetes mellitus (DM2) in developing countries remains unknown. The Brazilian government conducted a nationwide population screening program for type 2 diabetes mellitus (BNDSP) in which 22 million capillary glucose tests were performed in individuals aged 40 years and older. The objective of this study was to evaluate the life-time cost-effectiveness of a national population-based screening program for DM2 conducted in Brazil.

**Methods:**

We used a Markov-based cost-effectiveness model to simulate the long-term costs and benefits of screening for DM2, compared to no screening program. The analysis was conducted from a public health care system perspective. Sensitivity analyses were conducted to examine the robustness of results to key model parameters.

**Results:**

Brazilian National diabetes screening program will yield a large health benefit and higher costs. Compared with no screening, screen detection of undiagnosed diabetes resulted in US$ 31,147 per QALY gained. Results from sensitivity analyses found that screening targeted at hypertensive individuals would cost US$ 22,695/QALY. When benefits from early glycemic control on cardiovascular outcomes were considered, the cost per QALY gained would reduce significantly.

**Conclusions:**

In the base case analysis, not considering the intangible benefit of transferring diabetes management to primary care nor the benefit of using statin to treat eligible diabetic patients, CE ratios were not cost-effective considering thresholds proposed by the World Health Organization. However, significant uncertainty was demonstrated in sensitivity analysis. Our results indicate that policy-makers should carefully balance the benefit and cost of the program while considering using a population-based approach to screen for diabetes.

## Background

The socioeconomic burden of type 2 diabetes mellitus (DM2) is large and increasing. Early detection and treatment of DM2 seems a logical preventive action for several reasons. First, diabetes-related complications can occur before diabetes clinical diagnosis. Second, efficacy of early treatments in reducing complications is well established [1–8]. Third, acceptable and accurate screening tests are available [9, 10]. Importantly, recent evidence failed to demonstrate significant impact of screening asymptomatic individuals at increased risk for diabetes in reducing all-cause, cardiovascular, or diabetes-related mortality within 10 years [11]. However, a number of institutions have recommended opportunistic screening for high-risk individuals in certain circumstances [12–16]. Opportunistic screening, carried out at a time when people are seen, by healthcare professionals, for a reason other than the disorder in question, is one of the potential approaches to screening for DM2 [12].

Several countries have implemented opportunistic selective DM2 screening in high-risk populations [12]. By contrast, Brazil has implemented a massive population-based diabetes screening program.

The prevalence of diabetes in Brazil is high and represents one of the major challenges to the Brazilian publicly-funded National Healthcare System (SUS) [17]. In 2001, the Brazilian Ministry of Health implemented a public health strategy aiming at reorganizing diabetes and hypertension care delivery. The main aim of this strategy was to shift the focus of diabetes care from hospitals to primary care settings. A cornerstone of the strategy was the Brazil Nationwide Diabetes Screening Program (BNDSP), a one-time screening program conducted through primary healthcare services using fingerstick capillary blood glucose testing. Over 13,000 health care providers in approximately 40,000 SUS primary health care centers among Brazil’s 5507 municipalities were trained in diabetes diagnosis and management [18]. The BNDSP targeted 31 million adults aged 40 years or older who received health care through the SUS. Capillary blood glucose testing was conducted. With a massive participation rate, between March 6 and April 7, 2001, 22.1 million capillary blood glucose tests were performed. An initial assessment found that the program led to the diagnosis of approximately 345,000 new cases of diabetes at a cost of US$ 76 per case [19].

Determining whether to incorporate such a public health strategy into standard practice requires weighing the estimated benefits of population screening in reducing long-term complications against the long-term costs it generates. Following global recommendations for countries conducting screening strategies [12], the objective of this study is to evaluate the long-term cost-effectiveness of the BNDSP. Specifically, we estimated the lifetime costs and benefits of universal screening for type 2 diabetes compared to standard practice in Brazil, that is, no organized screening, taking the perspective of the Brazilian Public Healthcare System.

## Methods

We conducted a cost-effectiveness study in which a validated Markov model was populated with data from a national population-based screening program for DM2 conducted in Brazil, to evaluate the life-time costs and benefits of screening for DM2.

### Screening

During the BNDSP, a positive screening test was defined by a fasting capillary glucose ≥100 mg/dL or a casual glucose ≥140 mg/dL, and fasting was defined as absence of food ingestion for at least 4 h prior to capillary glucose test [18]. Sensitivity and specificity of capillary blood glucose screening considered in the model were 68 and 89 %, respectively [10]. These values were estimated considering the BNDSP participants reporting to be in fasting condition (47 %), for which the cut-off for positive test results was 100 mg/dL, and non-fasting condition (53 %), for which the cut-off was 140 mg/dL [19]. An additional fasting plasma glucose test and two extra physician visits were required for diagnostic confirmation of diabetes.

**Fig. 1:**
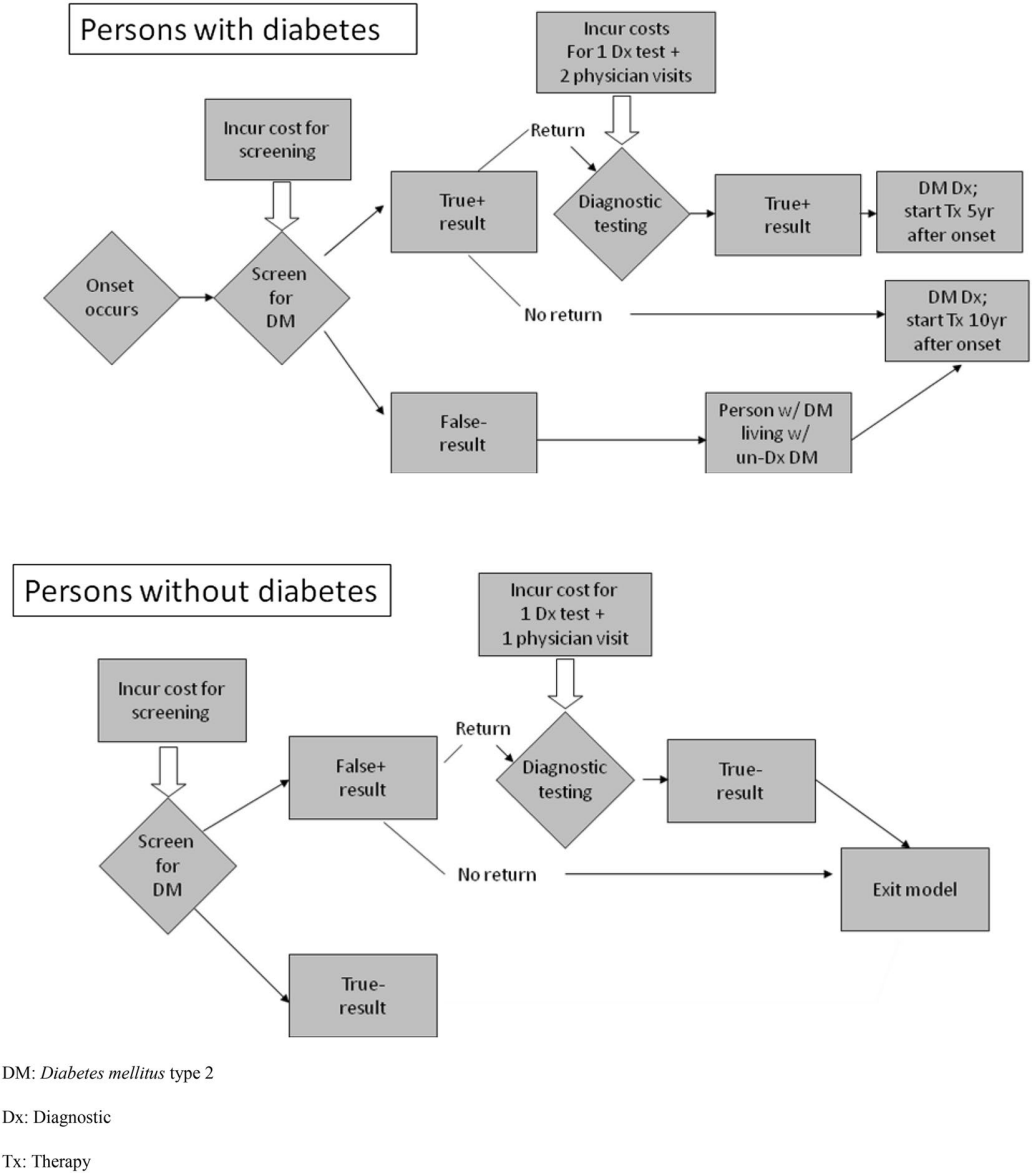
Progression of individuals in the screening module, Nationwide Population Screening Program for Diabetes. Brazil, 2001

**Fig. 2:**
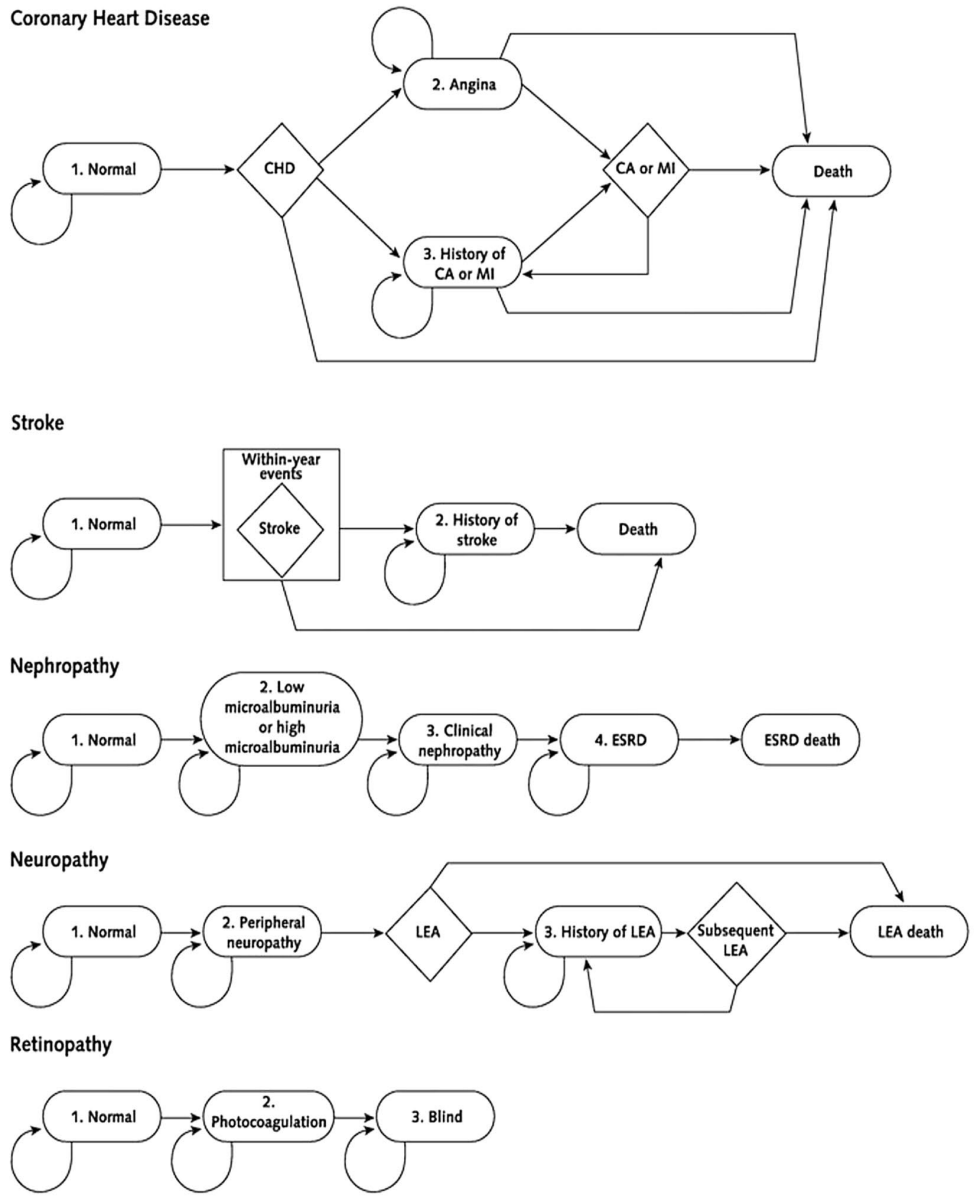
Markov model of diabetes disease progression

Universal DM2 screening as conducted in the BNDSP was compared to standard practice in Brazil at that time, that is, no organized screening. Progression of individuals with and without diabetes in the screening module is represented in Fig. 1. Individuals diagnosed with diabetes were assumed to enter the diabetes disease progression model (Fig. 2), described below.

### Post-screening interventions considered

During the BNDSP, specific recommendations were given regarding treatment. Diagnosed cases were managed aiming at blood pressure target of 135/80 mmHg and fasting serum glucose level of 110 mg/dL. Therefore, we assumed that intensified glycemic and hypertension treatment were promptly initiated at diagnosis. Based on the United Kingdom Prospective Diabetes Study (UKPDS), we assumed the intensified glycemic control consisted of one or more generic drugs (metformin, glibenclamide, and/or insulin) aiming at fasting serum glucose level of 110 mg/dL [1, 20]. The effect of intensified glycemic treatment is modeled as slowing progression of microvascular complications by reducing hemoglobin A_1c_, and thus lowering hazard rates for microalbuminuria, nephropathy, peripheral neuropathy, and photocoagulation [1]. No effects on macrovascular complications were assumed in the base case [1].

All persons with hypertension were assumed to receive standard hypertension treatment (targeting diastolic blood pressure of 90 mmHg) until they receive a diagnosis of diabetes, after which they receive intensified hypertension treatment (targeting diastolic blood pressure of 85 mmHg) [3] consisting of angiotensin converting enzyme inhibitor (captopril), β blocker (propranolol) or thiazides as single or combination therapy [2]. Model estimates of this approach assumed a 17–44 % relative risk reduction for stroke and 13 % risk reduction for CHD [2].

### Model

We used a modified version of the CDC/RTI type 2 diabetes cost-effectiveness simulation model to simulate the long-term health and economic consequences of the BNDSP. The CDC/RTI model is a Markov-based model developed by the Centers for Disease Control and Prevention and RTI International (CDC/RTI). The model simulates disease progression based on annual transition of multiple disease states. The outcomes include lifetime development of diabetic complications, diabetes-related health care costs, life years, and quality adjusted life years gained (QALYs) [21]. In progressing through the model from the onset of diabetes to death, people with diabetes can develop five types of complications: nephropathy, neuropathy, retinopathy, coronary heart disease (CHD), and stroke (Fig. 2). Each complication/health state has a corresponding health utility value ranging from 0 (death) to 1 (perfect health). The basic model structure and key model parameters have been previously described [21–23]. Annual transition probabilities for the health states considered in the model are presented in Table 1.

**Table 1:** Annual transition probabilities for health states considered in the model

Health state	Transition probability	Source
Normal to microalbuminuria		
Baseline	0.033	[1]
Hypertensive with moderate control	0.056	[48]
Hypertensive with tight control	0.038	[48]
Microalbuminuria to nephropathy		
Baseline	0.075	[2]
Hypertensive with moderate control	0.151	[48]
Hypertensive with tight control	0.128	[48]
Nephropathy to end-stage renal disease		
0–11 years since diabetes diagnosis	0.004	[49, 50]
12–19 years since diabetes diagnosis	0.039	[49, 50]
20–94 years since diabetes diagnosis	0.074	[49, 50]
Normal to peripheral neuropathy	0.0036	[1]
Peripheral neuropathy to lower-extremity amputation		
0–7 years since diabetes diagnosis	0.028	[51]
8–12 years since diabetes diagnosis	0.046	[51]
13–18 years since diabetes diagnosis	0.056	[51]
19–94 years since diabetes diagnosis	0.140	[51]
Normal to photocoagulation		
Baseline	0.011	[1]
Hypertensive with moderate control	0.017	[2]
Hypertensive with tight control	0.010	
Photocoagulation to blindness		
Baseline	0.107	[2]
Hypertensive with moderate control	0.107	[2]
Hypertensive with tight control	0.107	[2]
Normal to stroke	Framingham equation	
Stroke to death		
Immediate	0.142	[52]
1 year	0.092	[52]
Normal to CHD		
CHD(t) = [F(t) − F(t − 1)]/[1 − F(t − 1)]*		[53]

*CHD* coronary heart disease

* Probability of a new case of CHD at period t given by a Weibull function

The model includes a screening module, and assumes that in the absence of screening, diagnosis would occur 10 years after its onset while screening would reduce this pre-diagnosis interval by 5 years [24]. Progression of persons through screening and clinical diagnosis is shown in Fig. 2.

The CDC/RTI model has been used to assess the cost-effectiveness of screening for undiagnosed type 2 diabetes and pre-diabetes in the United States [22, 23]. For this study, we adjusted epidemiological and cost parameters using Brazilian data.

### Epidemiological data

Demographic and mortality data for the general population were obtained from the Brazilian National Institute for Geography and Statistics (IBGE) for 2002. Prevalence of risk factors for cardiovascular complications in the Brazilian population was based on surveys [25–27] (Table 2). Estimates of undiagnosed diabetes prevalence by age-groups and hypertension level were obtained from the BNDSP [28] (Table 2). Age and gender-specific estimates of the incidence of true diabetes in the population were obtained applying DISMOD II software [29] to data from a prevalence survey [30].

**Table 2:** Estimated prevalence of undiagnosed diabetes, smoking, hypertension and hypercholesterolemia in the Brazilian population

Age group (years)	Women (%)	Men (%)
Prevalence of undiagnosed diabetes [28]		
40–44	1.77	2.31
45–49	2.63	3.23
50–54	3.56	4.30
55–59	4.09	5.06
60–64	4.57	4.61
65–69	4.53	4.97
70–74	4.62	4.75
75 and older	4.79	4.53
Prevalence of smoking [25]		
18–34	11.8	19.2
35–49	20.8	25.5
50 and older	11.4	24.2
Prevalence of hypertension [26]		
35–44	17.9	15.3
45–54	31	28.7
55–64	47.2	37.7
65–74	57.5	52.8
75+	52	46.5

Age group (years)	Population (%)
Prevalence of hypercholesterolemia [27]	
Up to 24	8
25–34	10.9
35–44	20.9
45–54	28.8
55 and older	32.3

### Economic data

Four types of costs were considered: associated with diabetes screening, diabetes treatment, diabetes-related complications, and other medical care.

Costs associated with screening and confirmatory diagnosis have been previously estimated based on actual expenses of the BNDSP, which has been previously described [19]. A total of 22.1 million capillary glucose tests were performed, of which 16.4 % were positive. Total screening and diagnostic costs were US$ 26.19 million (Int$ 104.32 million), including national level costs of diagnostic material, social mobilization and media campaign, training of healthcare workers, and management costs. The screening cost per screened individual was estimated at US$ 1.16. Diagnostic confirmation costs were US$ 2.97 per each individual screened positive during the BNDSP.

The costs of glycemic control included three resource components: drug use, physician visits, and self-testing [31]. Treatment modalities considered were oral hypoglycemic agents only, insulin only, and both oral hypoglycemic agents and insulin. Intensified glycemic control considered initial treatment with metformin. The proportion using each drug regimen varied by duration of diagnosed diabetes and was estimated from the UKPDS [1, 32]. Daily cost of medications were estimated considering average market prices assuming standard doses. Incremental cost of intensive glycemic control (relative to standard control) was estimated per year, depending on the number of years since diagnosis.

The costs of standard and intensified hypertension control were estimated to reflect clinical practice in Brazil, where standardized drug regimens include the use of generic thiazides, propranolol, and captopril. We assumed that the maximum number of drugs that would be taken at any given time was three [33]. The incremental cost of intensive hypertension control, relative to standard control, was estimated per year, depending on the number of years since diabetes diagnosis.

Cost of diabetes-related complications included the cost of nephropathy, neuropathy, retinopathy, CHD, and stroke. One-time and annual costs of complications considered in the model (Table 3) were calculated. Healthcare resource utilization was estimated based on Brazilian guidelines, considering diagnosis and annual follow-up procedures for end stage renal disease, angina and stroke; one time diagnosis of peripheral neuropathy; and one time photocoagulation procedure. Each resource was multiplied by its unit costs, which for medical procedures, exams, hospitalization and medical visits considered SUS reimbursement values [31].

**Table 3:** Direct medical costs of diabetes complications in Brazil, 2001

Diabetes complication	Type of cost	Cost (2001 US$)
Nephropathy		
Clinical nephropathy	One time	267
End stage renal disease	Annual	9527
Neuropathy		
Peripheral neuropathy	One time	18
Lower extremity amputation	One time	309
Retinopathy		
Photocoagulation	One time	15
Coronary heart disease		
Angina	One time	776
	Annual	669
History of CA/MI	Annual	669
CA/MI death without hospitalization	One time	15
CA/MI death within 30 days with hospitalization	One time	368
CA/MI survivors	One time	776
Stroke		
Stroke	One time	955
	Annual	462
Immediate death from stroke	One time	180
Cost of death	One time	304

End-stage renal disease

*CA* cardiac arrest, *MI* myocardial infarction

One time angina and stroke costs were obtained from the National Information System on Hospitalizations in SUS (SIH), considering costs reimbursed by SUS for patients admitted to SUS in 2002 with diagnosis of the above conditions.

Direct cost of clinical nephropathy; lower extremity amputation; and death due to stroke or CHD, were obtained from follow-up data from all patients with each of these conditions admitted at the Hospital de Clínicas de Porto Alegre of the Federal University of Rio Grande do Sul (HCPA) during 2002.

Normal medical care costs that are not specific to diabetes care were estimated considering the Brazilian average government health expenditure/person (i.e., GNP per capita on health) of US$ 94 (Int$ 374) per year in 2002 [34]. Cost of death was estimated as a proportion of hospital expenditures prior to death [35], obtained from the cohort of all patients at the HCPA who progressed to death during 2002.

### Analyses

By estimating lifelong complications and death in a hypothetical population cohort, the model predicts the lifetime incidence of diabetes complications and QALYs for each true case of diabetes, considering utility values of the CDC/RTI model [21–23]. Per person change in life-years and QALY with screening were calculated, and the sum of all estimated costs and expected QALYs for each strategy considered in the analysis were used to calculate the incremental cost-effectiveness ratio of screening relative to no screening. All costs are presented in US dollars, considering the exchange rate in December 2001 (1 US$ = R$ 2.35). To allow for international comparison, we present results in both US$ and international dollars (Int$) considering year 2001/2002 purchasing power parity exchange rates (1 Int$ = R$ 0.59). All costs were converted to 2002 reais using the Consumer Price Index from the Brazilian Central Bank [36].

We took the perspective of the public health care system, as the costs of the screening program were paid by the publicly funded SUS and the population receiving the benefits of the strategies evaluated is covered by the SUS. A baseline 5 % discount rate was applied to future costs and QALYs [37].

### Sensitivity analysis

We conducted one way sensitivity analyses to investigate the effect of key parameter values and assumptions in cost-effectiveness ratios (Table 4).

**Table 4:** Sensitivity analysis

Base case	Incremental cost-effectiveness ratio (US$/QALY)
Base case	31,147
Detection benefit from screening	
4 years	34,927
6 years	27,005
Screening costs	
+20 %	31,636
−20 %	30,708
Incremental intensified glycemic treatment costs	
+20 %	33,558
−20 %	28,759
Incremental intensified hypertension treatment costs	
+20 %	31,083
−20 %	31,234
Complication costs	
+20 %	30,876
−20 %	31,442
Discount rates applied to costs and QALYs	
1 %	21,281
10 %	44,424
Utility weights associated with diabetes	
+20 %	26,874
−20 %	36,977
Effects of intensive glycemic control	
CHD risk reduction: 20 %	15,688
Stroke risk reduction: 20 %	28,029
CHD and stroke risk reduction: 20 %	14,769
Scenario Analysis	
Selective screening of screening of hypertensive individuals only	22,695
Control group not receiving intensive glucose and hypertension treatment	7505

We varied the costs of screening, intensified glycemic and hypertension therapy, and diabetes complications, as well utility weights associated with diabetes and its complications by ±20 %. The time assumed between diabetes onset and screening (detection benefit from screening) varied from 4 to 6 years, and discount rates varied from 1–10 %. Risk reduction of intensive glycemic control on macrovascular complications were varied to 20 % for both myocardial infarction and stroke, assuming that 50 % of the individuals newly diagnosed with diabetes would receive metformin, and considering risk reduction estimates from UKPDS 34, which showed a relative risk reduction of 41 % for stroke and 39 % for myocardial infarction [32]. We also estimated how the cost-effectiveness of the BNDSP would change by assuming persons with diabetes would not receive intensive glucose and hypertension control under the non-screening scenario, and screening would occur only in hypertensive individuals only during the BNDSP.

### Ethical considerations

This study has been carried out in accordance with the Declaration of Helsinki, and the study project was approved by the Ethics Committee of the Federal University of Rio Grande do Sul.

## Results

### Lifetime development of diabetic complications

Screening all adults aged 40 years or older decreased the incidence of all diabetes complications considered in the model and increased survival. Cumulative incidence was reduced from 0.49 % for non-screened population to 0.28 % for the screened for end stage renal disease, from 0.76 to 0.58 % for lower extremity amputation, from 8.4 to 7.4 % for stroke, from 33.8 to 29.6 % for CHD, and from 3.6 to 2.3 % for blindness. Screening leads to a slightly longer life expectancy, adding approximately 13 weeks to the average lifespan of those detected at screening.

### Diabetes-related health care costs, life-years and QALYs

Compared with no screening, population screening increases lifetime costs, primarily due to increased costs of introducing treatment 5 years earlier. Modeling shows that this cost of treatment for five additional years for those diagnosed was approximately 20 times the cost of screening.

Screening adults 40 years or older would increase the life-time costs by US$ 489 but results in a gain of 0.035 life-years and 0.0157 QALYs (Table 5). In the base-case analysis, the incremental cost-effectiveness ratio was estimated to be US$ 31,147 (Int$124,060) per QALY.

**Table 5:** Lifetime costs, life-years, QALYs and incremental cost-effectiveness per true case of diabetes diagnosed

	Cost (US$) (discounted)					Health outcomes (discounted)	Incremental cost-effectiveness ratio
	Cost of treatment	Cost of complications	Cost of screening	Costs of intensified glycemic and hypertension control	Total costs	Remaining QALYs	Total cost/QALY (US$)
No screening	3015	911	0	344.967	4271	4.6436	
Screening	3308	888	35.965	528.104	4760	4.6593	
Incremental	292.378	−22.478	36.965	183.137	489	0.0157	31,147

Brazilian nationwide population screening program for diabetes, 2001

### Sensitivity analyses

In sensitivity analyses, cost-effectiveness ratios were sensitive to several parameters (Table 4), including discount rates, detection benefit from screening, utility rates associated with diabetes, and cost of intensified glycemic control. On the other hand, varying screening cost, complication costs, and intensified hypertension control had minimal effect.

If benefit from early glycemic control on cardiovascular outcomes was assumed, i.e., when risk reduction of intensive glycemic control on macrovascular complications was considered 20 % for both myocardial infarction and stroke, cost-effectiveness ratio fell to US$14,769/QALY (Int$ 58,825/QALY). Similarly, if screening was conducted only among hypertensive individuals, the cost-effectiveness ratio decreased to US$22,695/QALY (Int$ 90,395/QALY). Assuming that non-screened individuals did not receive intensive glucose and hypertension controls after diabetes diagnosis, incremental cost-effectiveness ratios were considerably more favorable, estimated at US$ 7505/QALY gained (Int$ 29,893/QALY).

## Discussion

To our knowledge, this is the first cost-effectiveness study based on data of an actual population-based diabetes screening program. Additionally, our analysis was constructed by incorporating data from the screening program into an internationally recognized and validated model [21, 23].

Some specificities of Brazilian healthcare system should be considered when interpreting our results. The BNDSP occurred within the unique one of a national reorganization of primary care for diabetes. In this sense, screening was as much a strategy used to maximize mobilization as an isolated objective, and therefore several less tangible but equally important outcomes, not considered in this analysis, add to its benefits. These include the training of primary healthcare professionals in diabetes management, the increase in population awareness of diabetes as a significant health problem, the increase in access to healthcare services and the greater availability of drug therapy for individuals with diabetes and hypertension. If these aspects were considered, as mentioned above, the benefits of screening strategy would be higher.

In our base case analysis we did not consider cardiovascular benefit from early glycemic control for which evidence was not available at the time of the BNDSP; nor the use of statins in individuals diagnosed with diabetes and with high cholesterol levels, to reflect clinical practice in Brazil at the time of the BNDSP. We did, however, assumed that all individuals received intensified glycemic and hypertension treatment once diagnosed with diabetes, regardless of being diagnosed through screening or not. Considering such assumptions, our results were not cost-effective by WHO’s standards, which considers the cost-effectiveness threshold as up to 3 times the National Gross Domestic Product (GDP)/capita (US$ 9150 considering the 2002 Brazilian GDP per capita of US$ 3050). However, this recommended threshold is proposed for costs per disability life years (DALYs) and not QALYs, and has been criticized as having major shortcomings [38]. As recently presented in a publication discussing CE thresholds, cost–effectiveness analysis is useful only in the context of the choices available in a particular setting and context, and resulting CE ratios should be placed in the context of other, local policy and programme options, including funding sources. Other criteria for policy decisions, such as equity, ethics and political feasibility, should be taken into account when interpreting such results [38].

In sensitivity analysis, changes in the effect that is assumed for intensive glycemic control on CHD and stroke risk reduction significantly affected our results, with cost-effectiveness ratios as low as 14,769 US$/QALY. Though still a subject of considerable controversy, the current weight of the evidence suggests that glycemic control aiming for the UKPDS intervention group target, especially with metformin a first line treatment, may indeed protect against CHD and stroke [32, 39, 40]. However, as there is still controversy on the subject, we opted to be conservative and not consider such impact in the base case.

Another important finding in our sensitivity analysis was that higher screening program costs impacted little on overall cost-effectiveness. Screening costs have been shown to be significantly lower in low and middle income countries [12]. As compared to opportunistic screening, additional costs of population based screening programs delivered as mass-population preventive health interventions, such as immunization campaigns, may be justifiable if they reach higher participation rates and are able to provide additional benefits to the population.

It is important to acknowledge that the costs of drug therapy, medical procedures and services in Brazil are very low when compared to published medical care cost in high income countries. Evidence suggests that health care system reimbursement is lower than the actual costs of services [41]. As the costs of managing complications were greater than those of early intensified treatment of hyperglycemia and hypertension, our cost-effectiveness ratios may well be conservative ones.

Some limitations of our study should be acknowledged. First, we considered only one-time screening. Periodic, repeat screening would most likely yield less favorable cost-effectiveness ratios, as demonstrated by previous studies [23]. Second, the intangible benefits of the screening program mentioned above, which appear large, were not measured in our analyses. Last, we did not consider the use of statins in individuals diagnosed with diabetes and with high cholesterol levels. We opted not to because at the time the BNDSP was implemented, statins were not recommended due to their very high costs then. Nowadays, with lower costs of statins due to the availability of generics and their demonstrated effect in reducing macrovascular complications in diabetic individuals with high cholesterol levels, the recommendation of use of statins would result in more favorable cost-effectiveness ratios for a screening program similar to that implemented in Brazil.

A WHO expert group has recommended that countries define policies for diabetes diagnosis and treatment [12]. Various guidelines from high income countries recommend selective screening for high risk individuals [13–16]. Recent studies have documented that highly intensive glycemic control is not beneficial [42] and have failed to document that intensive treatment after screening [43] reduces cardiovascular events and mortality [44]. However, the interventions we modeled were less intensive and were compared to no screening.

Recent cost-effectiveness analyses of screening aimed to detect not diabetes, but rather those at high risk to develop the disease, have suggested that, in the long term, such screening, followed by an intensified program to promote and support lifestyle changes, may be not only cost-effective, but also cost saving [45]. If screening to detect those at high risk for diabetes is implemented, individuals with prevalent, previously undetected diabetes will inevitably be identified in the process. As the costs of initial preventive treatment in diabetes far exceed those of screening, population strategies aimed primarily at high risk individuals could make sense [45, 46] although a recent meta-analysis has raised the important issue of the effectiveness of community-based interventions [47].

New studies which evaluate screening benefits and costs to detect both diabetes and those at high risk to develop diabetes are necessary to clarify the cost-effectiveness of population screening strategies in today’s context.

Our findings are useful to any country considering alternatives for screening programs for the early diagnosis of diabetes. However, countries with different healthcare systems may find a significant difference in the benefits and costs of subsequent treatment of diabetes to prevent complications. In this regard, these results are generalizable only to countries with health care systems in which access to treatment and prevention of complications from diabetes is reasonably guaranteed.
